# Utility of Continuous Glucose Monitors for Improved Detection of Cystic Fibrosis‐Related Diabetes

**DOI:** 10.1002/ppul.71267

**Published:** 2025-08-26

**Authors:** Claire E. Moore, Wenya Chen, Stacy Bichl, Alexis Wong, Carolyn Heyman, Sarayu Ratnam, Monica E. Bianco

**Affiliations:** ^1^ Division of Endocrinology, Ann & Robert H. Lurie Children's Hospital of Chicago Chicago Illinois USA; ^2^ Department of Pediatrics, Ann & Robert H. Lurie Children's Hospital of Chicago Northwestern University Feinberg School of Medicine Chicago Illinois USA; ^3^ Stanley Manne Children's Research Institute Ann & Robert H. Lurie Children's Hospital of Chicago Chicago Illinois USA; ^4^ Division of Pulmonary Medicine Ann & Robert H Lurie Children's Hospital of Chicago Chicago Illinois USA

**Keywords:** continuous glucose monitoring, cystic fibrosis, cystic fibrosis‐related diabetes, oral glucose tolerance test, screening

## Abstract

**Background:**

Cystic fibrosis‐related diabetes (CFRD) can be associated with decline in pulmonary function and nutritional status. Earlier diagnosis of CFRD than offered by annual recommended oral glucose tolerance test (OGTT) and earlier initiation of insulin may help prevent clinical decline. This retrospective study investigates the utility of continuous glucose monitoring (CGM) for detection of hyperglycemia in patients with cystic fibrosis (CF).

**Methods:**

In this single‐center, retrospective study, we analyzed data from 18 patients with CF over age 10 who had an abnormal OGTT and subsequently had at least 24 h of CGM data. EasyGV software was used to calculate multiple measures of CGM variability. Differences in OGTT and CGM measures were explored across four glucose‐tolerance groups: indeterminate, fasting hyperglycemia, impaired glucose tolerance (with or without fasting hyperglycemia), and CFRD.

**Results:**

Multiple CGM measures correlated with components of the OGTT. Across glucose‐tolerance groups, significant differences were observed for the OGTT 2‐h glucose (*p* = 0.002), mean of daily differences from CGM (*p* = 0.03), and standard deviation from CGM (*p* = 0.02). Approaching significance was the lability index (*p* = 0.05) from the CGM data. Glucose management indicator (GMI), continuous overlapping net glycemic action (CONGA), glycemic risk assessment in diabetes equation (GRADE), and average daily risk range (ADRR) showed negative correlations with change in forced expiratory volume over 1 s (FEV1) over the year before OGTT.

**Conclusion:**

Markers of glycemic variability may be important variables distinguishing between degrees of abnormal glucose tolerance, including CFRD. This area warrants further research with a larger sample size.

## 1 Introduction

Cystic fibrosis (CF) is an autosomal recessive condition caused by various mutations in the transmembrane conductance regulator (CFTR) protein and impacts multiple organs, including the pancreas [1]. Cystic fibrosis‐related diabetes (CFRD) affects up to 20% of adolescents and up to 50% of adults with CF [2]. Its pathophysiology is multifactorial and incompletely understood. Early defects in insulin secretion lead to a loss of first‐phase insulin secretion [3, 4], while fasting hyperglycemia is a later finding in CFRD [5]. The role of the CFTR protein in the function of islet cells is unclear [6], but exocrine pancreatic damage is thought to contribute to beta cell loss and altered islet composition [7, 8]. Incretin secretion and insulin resistance also impact glycemic regulation in people with CF (PwCF) [6, 9].

Median survival of PwCF is increasing with widespread use of CFTR‐modulator therapy. CFTR‐modulator therapy results in improved protein and lung function [2]. However, the impact of this therapy on incidence and course of CFRD is unclear. Some studies suggest reduction in insulin requirements [10] or improvements in glucose time in range [11] while other data find lack of glycemic benefit [12, 13]. Because the impact of CFTR‐modulator therapy on CFRD remains unclear, early detection and treatment are still important to reduce the morbidity and mortality associated with this complicaton [14, 15]. Although the oral glucose tolerance test (OGTT) remains the current standard of care for annual CFRD screening starting at age 10 [16], it is a suboptimal screening test due to poor reliability from test to test, poor tolerability, and the fact that the test was not designed to assess the impact of hyperglycemia on PwCF [17].

Continuous glucose monitoring (CGM) could serve as an adjunctive or alternative screening or diagnostic tool for CFRD, but CGM diagnostic criteria do not yet exist. CGM sensors, worn for 7–14 days, measure interstitial glucose every few minutes. Studies have identified CGM parameters (e.g. interquartile range [18], percent time over 140 mg/dl [19], and glucose excursions above 200 mg/dl [19, 20, 21]) that correlate with the OGTT parameters.

Research to date suggests that clinically relevant dysglycemia (i.e. glucose peak over 200 mg/dl associated with poorer lung function [22] and percentage of time of 140 mg/dl associated with decline in weight standard deviation score [23]) can be identified on CGM even when OGTT results are normal. A 5‐year prospective study of 112 children and adults with CF found that increase in area under the curve > 140 mg/dl on CGM between two annual visits was associated with decline in lung function while variation in the 2‐h glucose on OGTT was not [24]. Notably, decline in forced expiratory volume in 1 s (FEV1), forced vital capacity (FVC), and body mass index (BMI) often precede CFRD diagnosis by OGTT [25, 26]. Insulin initiation can reverse these trends [26, 27], even in those with abnormal glucose tolerance but without CFRD [28]. Identifying CGM glycemic parameters linked to pulmonary and nutritional decline could help prevent these complications.

This retrospective study expands upon existing literature by investigating the full array of markers of glycemic variability measurable with CGM data. The study compares screening OGTT parameters and classification with CGM data to assess the utility of CGM to differentiate between degrees of glucose intolerance. Additional analysis focuses on correlations between both CGM and OGTT parameters and CF‐related clinical outcomes.

## 2 Materials and Methods

### 2.1 Study Subjects

This study was conducted at Ann and Robert H. Lurie Children's Hospital in Chicago, Illinois following institutional review board approval. It involved a retrospective review of data from the electronic health record and was thus exempt from the requirement of informed consent. Study subjects were patients aged 10 and older with CF but without prior diagnosis of CFRD. Starting in October 2021, the CF clinic at Lurie Children's offered CGM sensors to patients with CF who had abnormal OGTT results on their annual screen using a 75 g glucose load with glucoses collected at hours 0, 1, and 2. For patients who met criteria for CFRD with a fasting glucose ≥ 126 mg/dl or a 2‐h glucose ≥ 200 mg/dl, initiation of insulin occurred after the completion of both the OGTT and CGM. For inclusion in this study, patients had to have OGTT data and at least 24 h of CGM data between October 2021 and April 2024. Exclusion criteria included existing diagnosis of diabetes, age younger than 10 years old, history of transplant, use of medications (e.g. insulin, corticosteroids) that affect glycemia, pulmonary exacerbation requiring hospitalization within 90 days before OGTT date or CGM sensor placement. Inclusion and exclusion criteria were confirmed by chart review.

### 2.2 Study Design

Relevant demographic and clinical information was abstracted from the Epic electronic health record for eligible patients. Demographic information included date of birth, age at the time of the OGTT, sex, race, and ethnicity. Clinical information included CF‐related medications, HgbA1c, and OGTT data (fasting, 1‐h, and 2‐h glucose measures). The pulmonary markers included forced expiratory volume in 1 s (FEV1) and FEV1 percentile, and the nutritional markers included body mass index (BMI) and BMI percentile. These clinical values closest in time to the OGTT and closest to 1 year before the OGTT were abstracted.

All CGM data was obtained using Dexcom devices and was extracted from the Dexcom Clarity application. All CGM data was collected within 6 months following the date of OGTT with the exception of the data of one patient for whom the CGM was placed 8 months after the OGTT. Patients agreed to data sharing with their care team at the time of CGM sensor placement. Up to 72 consecutive hours of data was organized into comma‐separated value (CSV) files. To account for the CGM sensor warm‐up period and to standardize the timing of CGM across patients, the selected CGM data started at 6 AM on the second calendar day of CGM use. From these files, fasting, 1‐h postprandial, and 2‐h postprandial serum glucose values were inferred based on apparent rises in glucose values suggestive of meals and were abstracted manually. Percent time above 140 mg/dl and percent time above 180 mg/dl were calculated, and number of excursions over 200 mg/dl were tallied. EasyGV software was used to calculate multiple measures of CGM variability. These measures were m‐value, mean amplitude of glycemic excursions (MAGE), lability index, average daily risk range (ADRR), j‐index, low blood glucose index, high blood glucose index, continuous overall net glycemic action (CONGA), mean of daily differences, glycemic risk assessment in diabetes equation (GRADE), and mean average glucose. Glucose management indicator (GMI) was recorded, when available, from the Dexcom Clarity application.

The nature of the analysis was intended to be exploratory. The cohort was divided into four groups based on degree of glucose intolerance on the OGTT. The four groups of abnormal glucose tolerance in addition to the parameters for normal glucose tolerance are shown in Table 1. Due to the small sample size and the exploratory nature of the study, we considered impaired glucose tolerance with or without impaired fasting glucose to be one group.

**Table 1:** Classification of oral glucose tolerance categories in CF.

	Fasting	1‐h	2‐h
Normal	< 100 mg/dl	< 200 mg/dl	< 140 mg/dl
Indeterminate glucose tolerance	< 100 mg/dl	≥ 200 mg/dl	< 140 mg/dl
Impaired fasting glucose	100–125 mg/dl		
Impaired glucose tolerance			140–199 mg/dl
CFRD	≥ 126 mg/dl		≥ 200 mg/dl

The Shapiro‐Wilk test was used to assess normality of data. Normally distributed variables are summarized as mean and standard deviation (SD). Non‐normally distributed variables are summarized as the median (25th, 75th percentiles). Categorical variables are presented as n (%).

To compare the groups, we used the Kruskal‐Wallis test for continuous variables and the Fisher's Exact test for categorical variables. When an overall test was significant, we conducted pairwise, post‐hoc comparisons. P‐values were adjusted for multiple testing with the Holm–Bonferroni method. We calculated Pearson correlation coefficients to assess the relationships between OGTT and CGM parameters, CGM parameters and clinical outcomes, and OGTT parameters and clinical outcomes. A permutation test with 10,000 shuffles was used to test the significance of these correlations. Logistic regression analysis was performed on select CGM parameters to explore their ability to distinguish between CFRD and other degrees of abnormal glucose tolerance as determined by OGTT classification. A *p*‐value less than 0.05 was considered statistically significant for all analyses. Formal statistical adjustments for multiplicity were not included throughout the analysis. With the small sample size, application of multiplicity adjustments would have further inflated the risk of type II error and potentially increased the chance of missing effects worth future investigation. Statistical analyses were conducted using R version 4.1.0 within the RStudio environment.

## 3 Results

Eighteen patients met inclusion criteria. Baseline characteristics are shown in Table 2. There was no statistical difference in demographic characteristics among groups based on OGTT interpretation. Fifty‐six percent (*n* = 10) of the total patients were female. Mean age at time of OGTT was 15.4 years. Eighty‐three percent were prescribed CFTR modulator therapy. Four patients met criteria for diagnosis of CFRD based on OGTT results. Four patients had indeterminate glycemia (INDET), three had impaired fasting glucose (IFG), and seven had impaired glucose tolerance (IGT). By nature of the study criteria, no patients with normal OGTTs were included.

**Table 2:** Patient demographics.

	Total (*n* = 18)	Indeterminate (*n* = 4)	Impaired fasting glucose (*n* = 3)	Impaired glucose tolerance (*n* = 7)	CFRD (*n* = 4)	*p*
Sex, female	10 (56%)	1 (25%)	1 (33%)	5 (71%)	3 (75%)	0.42
Race						0.92
White/non‐Hispanic	12 (67%)	2 (50%)	2 (67%)	5 (71%)	3 (75%)	
White/Hispanic	2 (11%)	0 (0)	0 (0)	1 (14%)	1 (25%)	
Other/Hispanic	3 (17%)	1 (25%)	1 (33%)	1 (14%)	0 (0)	
White and Asian/non‐Hispanic	1 (6%)	1 (25%)	0 (0)	0 (0)	0 (0)	
Modulator use						0.79
None	3 (17%)	1 (25%)	0 (0)	1 (14%)	1 (25%)	
Trikafta	14 (78%)	3 (75%)	2 (67%)	6 (86%)	3 (75%)	
Study drug	1 (6%)	0 (0)	1 (33%)	0 (0)	0 (0)	
Age, years	15.4 ± 3.0	15.6 ± 3.8	15.8 ± 2.7	14.6 ± 3.6	16.2 ± 1.6	0.89
Change in BMI over one year, kg/m^2^	0.5 ± 1.0	0.4 ± 0.5	0.01 ± 0.9	0.5 ± 1.0	1.1 ± 1.5	0.51
Change in BMI percentile over one year, %	−1.1 ± 8.5	−2.6 ± 5.2	−8.5 ± 11.0	−1.3 ± 3.7	6.2 ± 12.0	0.31
Change in FEV1 over one year, liter	0.1 ± 0.2	0.1 ± 0.2	0.3 ± 0.1	0.1 ± 0.2	−0.03 ± 0.3	0.18
Change in percent predicted FEV1 over one year, %	−0.9 ± 5.8	1.0 ± 5.6	2.3 ± 1.5	−2.5 ± 5.1	−2.8 ± 8.8	0.39

*Note:* Data were reported as *n* (%) for categorical variables, mean ± SD for normally distributed continuous variables, and median (25th percentile, 75th percentile) for non‐normally distributed continuous variables.

Abbreviations: BMI, body mass index; CFRD, cystic fibrosis‐related diabetes; CGM, continuous glucose monitor; FEV1, forced expiratory volume in one second; GMI, glucose management indicator; HgbA1c, hemoglobin A1c; OGTT, oral glucose tolerance test.

The majority (*n* = 13) of patients had CGM data within 2 months of their OGTT date. The largest gap between OGTT and CGM collection occurred in one patient for whom the CGM placement occurred 8 months after the OGTT. The remainder of the OGTTs and CGM placements occurred within 6 months of each other. Four patients had under 72 h of CGM data, but all had at least 24 h. Three patients were missing GMI data.

Table 3 shows correlations between CGM and OGTT parameters. Statistically significant, positive correlations were noted between CGM mean fasting glucose and both OGTT fasting (*r* = 0.66) and 1‐h values (*r* = 0.59), coefficient of variation and both OGTT fasting (*r* = −0.48) and 2‐h values (*r* = 0.53), standard deviation from the CGM report and OGTT 2‐h glucose values (*r* = 0.67), excursions > 200 mg/dl and OGTT 2‐h glucose values (*r* = 0.58), GMI and OGTT 1‐h glucose values (*r* = 0.54), and lability index (which measures variability between means of three consecutive glucose values) and OGTT 2‐h glucose values (*r* = 0.51). Notably, percentage of time over 140 mg/dl and over 180 mg/dl and the remainder of the markers of glycemic variability on CGM did not correlate with OGTT parameters.

**Table 3:** Correlation of OGTT measures with CGM measures.

	Fasting glucose of OGTT	1‐h glucose of OGTT	2‐h glucose of OGTT
Mean fasting glucose of CGM	**0.66** *	**0.59** *	0.06
Mean 1‐h glucose of CGM	0.17	0.23	0.27
Mean 2‐h glucose of CGM	0.41	0.16	0.39
Number of excursions over 200 mg/dL	0.30	0.39	**0.58** *
GMI	0.36	**0.54** *	0.44
Percentage of glucose values over 140 mg/dL	0.30	0.39	0.40
Percentage of glucose values over 180 mg/dL	−0.05	0.45	0.17
M value	−0.21	0.03	0.03
MAGE	−0.06	0.22	0.41
Lability index	−0.05	0.18	**0.51** *
Average daily risk range	−0.02	0.35	0.36
JI	−0.08	0.26	0.15
Low blood glucose index	−0.37	−0.41	0.06
High blood glucose index	−0.14	0.18	0.18
Continuous overlapping net glycemic action	0.05	0.40	0.13
Mean of daily differences	−0.13	0.21	0.40
Glycemic risk assessment in diabetes equation	0.04	0.34	0.15
Mean absolute glucose	−0.19	−0.25	−0.11
Coefficient of variation	**−0.48** *	−0.24	**0.53** *
Standard deviation from the CGM reports	−0.20	0.14	**0.67** **

* Denotes *p*‐value < 0.05.

** Denotes *p*‐value < 0.01.

Abbreviations: GMI, glucose management indicator; JI, a measure of glycemic variability that takes into account the mean and standard deviation of data; MAGE, mean amplitude of glycemic excursions; *M* value, a measure of glycemic variability and mean glycemia that is equivalent to the mean of the logarithmic transformation from the deviation of six standardization.

Associations between glycemic measures and OGTT interpretation are shown in Table 4. HgbA1c, OGTT fasting glucose, and OGTT 1‐h glucose were not statistically different across the four glucose tolerance groups. In contrast, OGTT 2‐h glucose was significantly different across groups (*p* = 0.002). Post‐hoc analyses revealed that the impaired fasting glucose group had significantly different 2‐h glucose than each of the other three groups (all Holm‐adjusted *p* < 0.05), whereas the other pairwise comparisons were not significant. For the CGM parameters, we found a significant overall difference in the mean of daily differences across the groups (*p* = 0.03). However, none of the pairwise tests remained significant after Holm‐Bonferroni adjustment. In addition, standard deviation from the CGM reports was different across the groups (*p* = 0.02); pairwise comparisons indicated a significant difference only between the impaired fasting glucose group and the CFRD group (Holm‐adjusted *p* = 0.03). Lability index and coefficient of variation were approaching statistical significance (*p* = 0.05).

**Table 4:** Glycemic measures.

	Total (*n* = 18)	Indeterminate (*n* = 4)	Impaired fasting glucose (*n* = 3)	Impaired glucose tolerance (*n* = 7)	CFRD (*n* = 4)	*p*
HgbA1c, %	5.6 ± 0. 6	5.6 ± na	5.1 ± 0.1	6.0 ± 0.6	5.5 ± 0.7	0.38
OGTT fasting glucose	100.8 ± 14.4	87.00 ± 6.5	105.3 ± 1.5	103.6 ± 11.2	106.3 ± 23.5	0.15
OGTT 1‐h glucose	210.9 ± 46.4	224.5 ± 20.2	187.3 ± 14.6	209.6 ± 61.4	217.3 ± 57.0	0.60
OGTT 2‐h glucose	154.6 ± 44.4	112.0 ± 17.9	111.3 ± 12.1	162.0 ± 9.7	216.8 ± 31.1	**0.002**
CGM fasting glucose	109.8 ± 18.2	98.7 ± 18.0	118.5 ± 10.6	112.0 ± 18.5	109.8 ± 22.9	0.50
CGM 1‐h glucose	180.1 ± 36.4	154.0 ± 28.8	171.5 ± 26.2	190.6 ± 46.3	185.5 ± 23.2	0.51
CGM 2‐h glucose	126.8 ± 24.5	95.0 ± 7.8	128.0 ± 8.5	136.6 ± 25.1	133.0 ± 19.6	0.07
Excursions over 200 mg/dl	3.5 (1, 6)	1 (0, 3)	5 (2.5, 5.5)	2 (1.5, 6)	8.5 (5, 11.5)	0.29
GMI, %	6.3 ± 0.4	6.3 ± 0.4	6.0 ± 0.5	6.3 ± 0.2	6.6 ± 0.5	0.59
% time > 140 mg/dl	19.0 (11.5, 30.4)	12.05 (10.5, 14.4)	25.1 (12.7, 28.2)	19.8 (15.2, 49.3)	39.9 (23.7, 53.2)	0.36
% time > 180 mg/dl	8.4 (2.8, 16.8)	1.9 (1.2, 9.9)	7.3 (3.7, 8.9)	13.4 (4.0, 38.8)	12.6 (8.1, 16.1)	0.40
M value	2.2 (1.3, 3.4)	1.7 (1.6, 2.0)	1.7 (1.4, 3.7)	1.8 (1.0, 3.1)	3.7 (3.3, 4.3)	0.27
MAGE, mg/dL	3.7 (3.4, 4.3)	3.4 (3.3, 3.6)	4.1 (3.1, 4.2)	3.5 (3.2, 3.7)	5.5 (4.9, 5.8)	0.12
Lability index	3.3 (2.5, 4.1)	3.6 (3.1, 3.9)	3.1 (2.0, 3.4)	2.5 (2.3, 3.2)	5.6 (4.8, 6.6)	0.05
Average daily risk range						0.16
Low risk	15 (83%)	4 (100%)	3 (100%)	6 (86%)	2 50%	
Moderate risk	2 (11%)	0 (0)	0 (0)	0 (0)	2 50%	
High risk	1 (6%)	0 (0)	0 (0)	1 (14%)	0 (0)	
JI	22.8 (19.5, 29.4)	20.4 (18.8, 23.0)	24.1 (17.5 25.8)	21.9 (21.1, 27.9)	30.5 (27.2, 32.1)	0.51
LBGI	1.6 ± 1.4	1.4 ± 0.8	1.3 ± 2.0	1.6 ± 1.7	1.9 ± 1.3	0.72
HBGI	2.5 (1.8, 3.6)	1.8 (1.7, 2.1)	2.5 (1.4, 2.5)	2.0 (1.9, 3.3)	3.8 (3.6, 4.1)	0.11
CONGA	5.8 (5.4, 6.5)	5.5 (5.2, 5.8)	5.9 (5.1, 6.3)	5.9 (5.6,7.0)	6.2 (5.6, 6.7)	0.72
MODD	1.4 (1.3, 1.6)	1.4 (1.3, 1.5)	1.0 (1.0, 1.2)	1.3 (1.3, 1.5)	2.2 (2.0, 2.4)	**0.03**
GRADE	2.1 (1.2, 3.1)	1.6 (1.3, 2.24)	2.2 (1.3, 2.7)	1.8 (1.4, 4.5)	3.2 (2.4, 4.0)	0.84
Mean absolute glucose	3.4 ± 0.6	3.8 ± 0.9	3.1 ± 0.5	3.2 ± 0.5	3.5 ± 0.3	0.36
Coefficient of variation	22.6 ± 4.1	23.3 ± 1.2	19.0 ± 2.4	21.0 ± 3.2	26.9 ± 4.8	0.05
Standard deviation from the CGM reports	28.4 ± 5.7	28.8 ± 5.0	22.7 ± 6.7	26.3 ± 2.3	35.3 ± 2.4	**0.02**

*Note:* Data were reported as *n* (%) for categorical variables, mean ± SD for normally distributed continuous variables, and median (25th percentile, 75th percentile) for non‐normally distributed continuous variables. Bold indicates significant correlations.

Abbreviations: CFRD, cystic fibrosis‐related diabetes; CGM, continuous glucose monitor; CONGA, continuous overall net glycemic action; GRADE, glycemic risk assessment diabetes equation; GMI, glucose management indicator; HgbA1c, hemoglobin A1c; HBGI, high blood glucose index; JI, a measure of glycemic variability that takes into account the mean and standard deviation of data; LBGI, low blood glucose index; MAGE, mean amplitude of glycemic excursions; MODD, mean of daily differences; *M* value, a measure of glycemic variability and mean glycemia that is equivalent to the mean of the logarithmic transformation from the deviation of six standardization.

Table 5 demonstrates correlations between glycemic measures and both change over year before the OGTT in % predicted FEV1 and change in BMI percentile. The results suggest that GMI (*r* = −0.63), CONGA (*r* = −0.51), GRADE (*r* = −0.51), and ADRR (*r* = −0.50) were significantly correlated with change in % predicted FEV1. No OGTT values correlated with clinical outcomes as shown in Table 6.

**Table 5:** Correlation of CGM measures with clinical change.

	Change over one year in % predicted FEV1	Change over one year in BMI percentile
Mean fasting glucose of CGM	−0.46	−0.23
Mean 1‐h glucose of CGM	−0.38	−0.07
Mean 2‐h glucose of CGM	−0.35	−0.01
Number of excursions over 200 mg/dL	−0.37	−0.05
GMI	**−0.63** *	0.10
Percentage of glucose values over 140 mg/dL	−0.47	0.02
Percentage of glucose values over 180 mg/dL	−0.27	0.02
Low blood glucose index	0.12	0.22
High blood glucose index	−0.36	0.08
Continuous overlapping net glycemic action	**−0.51** *	−0.03
Mean of daily differences	−0.35	0.06
Glycemic risk assessment in diabetes equation	**−0.51** *	0.01
Mean absolute glucose	0.17	0.24
M value	−0.26	0.08
MAGE	−0.32	0.04
Lability index	−0.33	0.06
Average daily risk range	**−0.50** *	0.21
JI	−0.42	0.001
Coefficient of variation	0.13	0.26
Standard deviation from the CGM reports	−0.22	0.19

* Denotes *p*‐value < 0.05.

Abbreviations: GMI, glucose management indicator; JI, a measure of glycemic variability that takes into account the mean and standard deviation of data; MAGE, mean amplitude of glycemic excursions; *M* value, a measure of glycemic variability and mean glycemia that is equivalent to the mean of the logarithmic transformation from the deviation of six standardization.

**Table 6:** Correlation of OGTT measures with clinical change.

	Change over one year in % predicted FEV1	Change over one year in BMI percentile
Fasting glucose	−0.36	0.11
1‐h glucose	−0.22	0.02
2‐h glucose	−0.14	0.28

*Denotes *p*‐value < 0.05.

Logistic regression analyses showed that none of the glycemic measures, including % time > 140 mg/dl, % time > 180 mg/dl, glucose excursions over 200 mg/dl, mean CGM glucose, HgbA1c, mean of daily differences, and lability index, were significantly associated with the odds of having CFRD.

## 4 Discussion

This study is the first study to report on the full array of markers of glycemic variation, and the findings highlight a possible role for glycemic variability in the diagnosis of CFRD. In this study, lability index, number of excursions over 200 mg/dl, coefficient of variation, and standard deviation on CGM reports correlated with OGTT 2‐h glucose, which is currently the primary diagnostic criterion for CFRD on the OGTT. While fasting glucose > 126 mg/dl can also establish the CFRD diagnosis, in our study, fasting glucose did not correlate with OGTT interpretation of CFRD, which suggests that fasting hyperglycemia is a later finding in CFRD and a less consistent feature in newly diagnosed cases of CFRD. Lability index also approached significance for positive association with OGTT determination of CFRD. Mean of daily differences, which is the average of the difference between values on different days at the same time, and CGM standard deviation did meet statistical significance between the OGTT‐defined CFRD and non‐CFRD groups.

Additionally, these data show CGM parameters may be helpful in identification of glycemic patterns associated with clinical pulmonary decline. In this study, GMI, CONGA, GRADE, and ADRR correlated with change in FEV1 over the year prior. None of the CGM parameters correlated with change in BMI over the year prior.

The most promising research to date toward establishment of CFRD criteria by CGM comes from Scully et al. and highlights time above range (3.4% time above 180 mg/dl and 17.5% time > 140 mg/dl) as highly sensitive and specific for identification of CFRD in 77 adult PwCF [29]. However, Chan et al. found these parameters to be less sensitive and specific when applied to 85 patients, including a substantial pediatric and adolescent population [30]. Both studies did include measures of glycemic variability (standard deviation, coefficient of variation, MAGE, and in the case of Scully, CONGA) [29, 30]. This study did not find significant correlations using time above range; however, small sample size may have limited this analysis.

In 2018, Chan et al. published novel findings that demonstrate that markers of glycemic variability have clinical significance with respect to lung function. CGM measures of peak glucose, excursions > 140 mg/dl, % time > 140 mg/dl, standard deviation, and MAGE were higher in youth with CF with normal glucose tolerance than in healthy controls. MAGE was among the parameters that correlated with decline in FEV1% and FVC% in the year prior [31]. Further studies focused on the full array of markers of glycemic variability are warranted.

Glucose excursions > 200 mg/dl have been explored as a potential diagnostic criterion for CFRD. In our study, glucose excursions > 200 mg/dl correlated with OGTT 2‐h glucose but was not associated with OGTT interpretation of CFRD. Kumar et al., using data from 19 studies, determined that the relative risk of an arbitrary CGM diagnosis of CFRD using a single excursion > 200 mg/dl when compared to the OGTT diagnosis was 2.92. Authors determined that a single excursion > 200 mg/dl was thus not an appropriate method of CFRD diagnosis as it resulted in many false positive results [32].

These findings did confirm lack of association between HgbA1c and OGTT‐defined CFRD. However, of interest, GMI, which is considered a clinical proxy for HgbA1c, did correlate with OGTT 1‐h glucose and was one of the few glycemic measures that correlated with decline in % predicted FEV1. CGM parameters, like GMI, may provide a path toward the development of screening methods to detect hyperglycemia that predicts complications of CFRD.

The ultimate goal in developing new CFRD criteria is to establish criteria that predict clinical outcomes. However, establishing alternative parameters that could reliably distinguish OGTT‐defined CFRD from normoglycemia and gradations of abnormal glucose tolerance is worthwhile as such parameters could be used in an adjunctive capacity to the OGTT. Adherence to OGTT screening recommendations in adolescence remains poor with rates only a little over 60% [33]. The necessity for an extended lab visit with multiple venous blood samples, the need to fast, and the side effects of the glucola (e.g. nausea, hypoglycemia) are undesirable aspects of the OGTT. By comparison, CGM sensors are generally well tolerated.

Several studies have started to explore OGTT‐sparing diagnostic approaches. Kutney et al. explored the reproducibility of an at‐home mixed meal tolerance test and found that parameter > 17.5% of time > 140 mg/dl and 3.4% of time > 180 mg/dl, 19 of 20 subjects had concordant findings across two CGM sensor wears [34]. Recently, outside of the realm of CGM research, data have supported use of homeostasis model assessment indices (HOMA), which take into account beta cell function and insulin sensitivity, as a pre‐OGTT screen [35, 36].

Consideration of age‐specific CGM criteria may be necessary. As highlighted by the conflicting results of the Scully and Chan studies, the course of progression to CFRD may be different for pediatric, adolescent, and adult populations. Declerq et al. studied 26 children and 21 adults who wore CGM sensors at night. AUC > 140 mg/dl and % time > 140 mg/dl at night were associated with lower FEV1 in adults but not in children [37].

As previously stated, the nature of this analysis was intended to be exploratory, and caution is exercised in interpreting results. Limitations of this study include small sample size, which affected analysis between glycemic groups and may have contributed to the lack of significance found. In particular, the sample size was too small to perform logistic regressions between each OGTT classification and the select CGM parameters. This area of analysis would be of particular interest as prior research has suggested that INDET may predict decline in FEV1 [11] and that patients with both IGT and INDET are more likely to develop CFRD than with either classification alone [38]. In addition to small sample size, the relatively short duration of the CGM wear and the time gap between the OGTT and the CGM placement (greater than 2 months but less than 8 months for five of the patients) are notable limitations. Future prospective studies would benefit from focusing on maximizing CGM wear and conducting OGTTs and CGMs concurrently. Another limitation of this retrospective study was that institutional practice was to offer CGMs only to patients with history of abnormal OGTTs. Therefore, data from patients with normal glucose tolerance on OGTT were not available for analysis. However, future studies would benefit from inclusion of patients with normal glucose tolerance to further understanding of how CGM parameters compare to the full range of glucose tolerance as defined by OGTT. Finally, the lack of dietary history was also a limitation of this retrospective study. When abstracting fasting, 1‐h, and 2‐h glucose values from CGM data, these values were only collected in cases of clear CGM peaks, indicative of meals. Where there were stretches with minimal upward variation in data, meals could not be inferred even over large stretches of time during which presence of a meal would be likely. This inability to identify mealtimes led to limited amount of CGM data regarding fasting, 1‐h, and 2‐h glucose values.

## 5 Conclusion

This study assessed relationships between CGM and OGTT parameters to investigate if CGM parameters could reliably detect differences in glucose intolerance seen on OGTT and if CGM parameters correlated with OGTT values. In this analysis, mean of daily differences and standard deviation, markers of glycemic variability from CGM data, were significantly different between participants with indeterminate, impaired fasting glucose, impaired glucose tolerance, and CFRD. CGM measures of mean glucose, coefficient of variation, standard deviation, glucose excursions > 200 mg/dl, GMI, and lability index did correlate with components of the OGTT. New correlations between CGM parameters and clinical outcomes were identified, but correlations seen in prior studies were not reproduced in our sample. Small sample size was a limiting factor. Multicenter, prospective research is needed to look more closely at the reliability of markers of glycemic variability in distinguishing between degrees of glucose intolerance on the OGTT as well as at predicting clinical decline.

## Author Contributions

**Claire E. Moore:** writing – original draft, funding acquisition, investigation, methodology, writing – review and editing, project administration. **Wenya Chen:** writing – review and editing, formal analysis, methodology. **Stacy Bichl:** data curation, writing – review and editing. **Alexis Wong:** writing – review and editing, data curation. **Carolyn Heyman:** writing – review and editing, data curation. **Sarayu Ratnam:** writing – review and editing. **Monica E. Bianco:** conceptualization, investigation, funding acquisition, writing – original draft, methodology, writing – review and editing, project administration, supervision.

## Ethics Statement

This study was conducted at Ann and Robert H. Lurie Children's Hospital in Chicago following approval of IRB 2024‐6730 on February 7, 2024. Support for this study was obtained through the Stanley Manne Children's Research Institute.

## Conflicts of Interest

The authors declare no conflicts of interest.

## Data Availability

The data that support the findings of this study are available from the corresponding author upon reasonable request.
